# Performance of a Matrix-Assisted Laser Desorption/Ionization Time-of-Flight Mass Spectrometry Testing Algorithm for the Rapid Identification of Clinical Filamentous Molds

**DOI:** 10.3389/fcimb.2022.915049

**Published:** 2022-06-15

**Authors:** John A. Fissel, Carrie K. Holdren-Serrell, Warda Memon, Sean X. Zhang

**Affiliations:** ^1^ Division of Medical Microbiology, Department of Pathology, The Johns Hopkins University School of Medicine, Baltimore, MD, United States; ^2^ Department of Pathology and Laboratory Medicine, Children’s Hospital Los Angeles, Los Angeles, CA, United States; ^3^ Microbiology Laboratory, The Johns Hopkins Hospital, Baltimore, MD, United States

**Keywords:** matrix-assisted laser desorption/ionization time-of-flight mass spectrometry, filamentous fungi, rapid identification algorithm, clinical mycology, mold diagnostics

## Abstract

One of the most significant challenges in the treatment of fungal infections is the relatively long turnaround time (TAT) required for fungal species identification. The length of TAT to identification can impact patient clinical outcomes by delaying appropriate targeted therapy. Matrix-Assisted Laser Desorption/Ionization Time-of-Flight Mass Spectrometry (MALDI-TOF MS) has demonstrated exceptional utility in the rapid identification of bacteria and yeasts in the clinical microbiology laboratory. The capability of MALDI-TOF MS for rapid identification of clinical isolates presents an opportunity for significant advancement in the identification of filamentous molds. In this study, we employed a diagnostic algorithm using MALDI-TOF MS for the rapid identification of filamentous molds in order to assess the impact of this technology on TATs. The majority of isolates included in this study were able to be identified by MALDI-TOF MS (78%). Further, these isolates were identified in less than three days from first detection of colony growth. This study demonstrates the utility of MALDI-TOF MS in the rapid identification of filamentous molds in the clinical mycology laboratory.

## Introduction

As our healthcare system faces a growing number of immunocompromised patients, there is a growing need for the rapid diagnosis of infectious fungal diseases (Pfaller and Diekema, 2007; Erjavec et al., 2009; Zhang et al., 2021) since this patient population is at high risk of fungal infections and severe disseminated mycoses (Bongomin et al., 2017; Terrero-Salcedo and Powers-Fletcher, 2020). Turnaround times (TATs) have a huge impact on patient clinical outcomes as there is a greater risk of mortality associated with longer time to diagnosis for systemic mycoses (Erjavec et al., 2009; Kozel and Wickes, 2014; Wickes and Wiederhold, 2018). In order to mitigate the risk of these severe outcomes, rapid diagnosis of fungi is critical for clinical mycology laboratories. MALDI-TOF MS presents a platform with the potential to rapidly identify filamentous molds.

**Figure 1:**
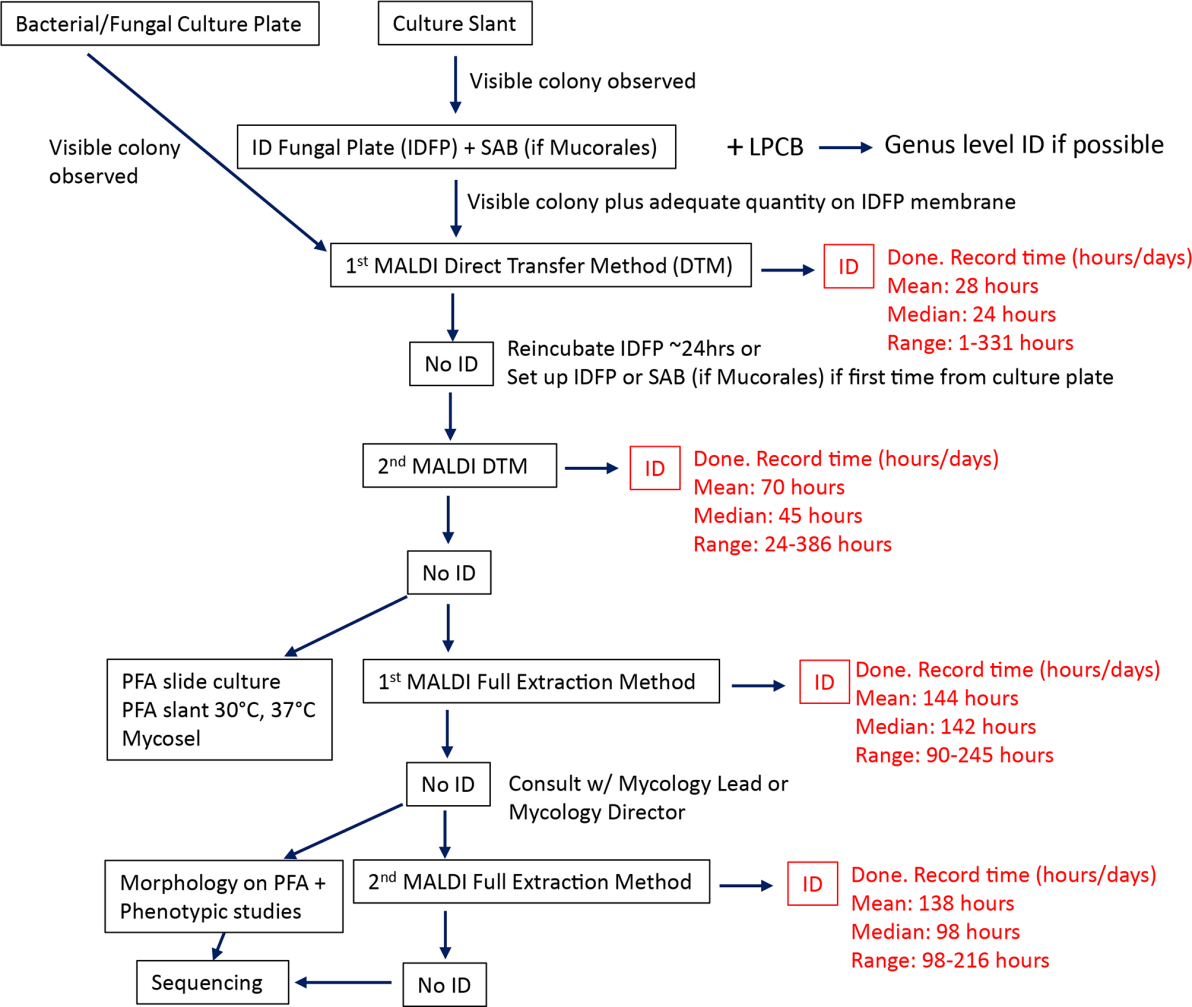
Rapid MALDI-TOF MS identification algorithm. Traditional mycology workup is supplemented by two rounds of MALDI-TOF MS using the direct transfer method followed by two more attempts of using MALDI-TOF MS broth full extraction method. The mean, median, and range provided for each MALDI-TOF step is based on the results of this study. SAB, Sabouraud agar; PFA, Potato flake agar; LPCB, Lactophenol Cotton Blue wet mount.

MALDI-TOF MS has revolutionized the identification of bacteria and yeasts by providing highly accurate identifications with relatively short TATs, compared to traditional methods (Patel, 2015). However, similar advancement in the identification of molds has lagged behind due to database limitations as a result of phenotypic heterogeneity of molds and inconsistent culture methods used to minimize heterogeneity and improve reproducibility (Sanguinetti and Posteraro, 2017; Lau et al., 2019; Patel, 2019). Our study utilizes a laboratory algorithm, leveraging MALDI-TOF MS for the rapid identification of filamentous molds. This approach includes four rounds of MALDI-TOF MS embedded into a traditional mycology workup algorithm (**Figure 1**). The first two rounds of MALDI-TOF MS utilize a direct transfer method (DTM), the second two rounds of MALDI-TOF MS utilize a broth culture and extraction method. The advantage of DTM is the speed with which results can be generated. Once sufficient biomass has grown on a solid agar plate or slant media, identification can be made in less than an hour. However, since fungi can have varying growth phases on solid media, proteins extracted from this method tend to be more heterogenous and produce varying results compared to broth culture methods (Zhang et al., 2021). To identify organisms that fail to be identified by DTM, a broth culture method is included in the algorithm. While broth culture adds additional culture time, it produces more homogenous cultures of hyphal structures that are well suited for more consistent MALDI-TOF MS spectra (Zhang et al., 2021).

## Materials and Methods

### Extraction and MALDI-TOF MS (Bruker MALDI Biotyper)

For the Direct Transfer Method the Bruker solid media extraction method was used. Briefly, a pure filamentous colony is selected from primary culture and MALDI-TOF MS is performed. If the organism is not identified or the identification has not been validated, then the isolate is used to make a lawn on ID Fungi Plates (IDFP) (Conidia, Quincieux, France) or Sabouraud dextrose agar (SABG) for Mucorales. These plates are incubated at 30°C for up to 72h until sufficient visible growth is observed. Hyphae are scraped from the IDFP membrane or from the edges of the SABG plates with a wooden toothpick and spotted directly on MALDI-TOF steel plate. Clinical isolates are then overlaid with 70% formic acid, allowed to dry, then overlaid with matrix. For the broth full extraction method, the Bruker liquid media extraction method was used. Briefly, a pure filamentous colony is selected, inoculated into Sabouraud dextrose broth and incubated at room temperature for 24-72h on a rotating incubator. Hyphae are collected from broth cultures, washed in reagent grade water two times, washed once with 75% ethanol, the pellet is obtained and dried. Next, the pellet is resuspended in 70% formic acid before the addition of an equal volume of 100% acetonitrile. The sample is again centrifuged and 1µl of the supernatant is spotted onto MALDI-TOF steel plate, allowed to dry, then overlaid with matrix. MALDI-TOF MS is performed using the Bruker MALDI Biotyper RUO Filamentous Fungal Library (version 3.0). For either method isolates with MALDI-TOF scores greater than or equal to 1.7 were considered identified. If the score was less than 1.7, the study MALDI-TOF algorithm (**Figure 1**) determined additional workup.

### Traditional Mycology Methodology

Initially macroscopic morphology is recorded for all colonies growing on primary plates/slants inoculated with specimen prior to microscopic examination *via* lactophenol cotton blue staining (LPCB). If possible, genus level identification is determined at this step before being sent for MALDI-TOF MS. If DTM MALDI-TOF MS is unsuccessful, then slide cultures are setup on PFA plates for these isolates and incubated at 30°C. If identification is not possible following first round of MALDI-TOF MS broth full extraction method, then additional phenotypic testing is performed on these isolates. Any isolates unidentified at this point are sequenced.

### Sequencing

Isolates that failed to be identified by both MALDI-TOF MS and phenotypic methods are sequenced *via* Sanger sequencing (Applied Biosystems 3500 Genetic Analyzer) for the internal transcribed spacer (ITS) and 26-28s rDNA (D1/D2) gene targets (Kwiatkowski et al., 2012). Briefly, a hyphal mass is harvested from the outer edge of the colony from a solid agar medium and DNA is extracted using Zymo Research Fungal/Bacterial DNA Kit, following kit instructions. Amplification PCR of ITS and D1/D2 targets are performed and resulting products are purified using microcon-100 microconcentrator columns (Merck-Millipore). Cycle sequencing is performed and extension products are purified using Qiagen DyeEx 2.0 Spin Kit. Sequencing data is analyzed using SmartGene software and database (SmartGene, Inc.) and National Center for Biotechnology Information (NCBI) database.

## Results

From December, 7^th^ 2020 to March 31^st^ 2021, 134 clinical filamentous molds were included in the rapid MALDI-TOF MS identification algorithm (**Figure 1**). Hyaline hyphomycetes were the majority (63%) of the organisms identified during that period (**Table 1**). The remaining organisms that were identified during the study were dermatophytes (16%), dematiaceous fungi (12%), mucorales (6%), basidiomycetes (2%), and a single dimorphic fungus (1%).

**Table 1:** Categorization of organisms included in study.

Organism Category	No. (%)
Hyaline hyphomycetes	85 (63)
Dermatophytes	22 (16)
Dematiaceous	16 (12)
Mucorales	8 (6)
Basidiomycetes	2 (2)
Dimorphic	1 (1)
Total	134

Overall, MALDI-TOF was effective in the identification of 78% (104/134) of the organisms that were encountered during the study period. Organisms that were able to be identified by MALDI-TOF MS using the direct transfer method (DTM) were identified on average at hour 28 and hour 70 for first and second DTM attempts, respectively. A majority of the organisms in this study were identified at the first DTM MALDI attempt (55%, 74/134). Organisms that were identified by MALDI-TOF using broth full extraction method took considerably longer to identify, on average about 138-144 hours, than organisms identified by DTM, owing to the increased time for broth cultures to grow. Identification by slide culture and phenotypic methods took approximately 96 hours for identification. Finally, organisms that ended up with sequencing identification took about 381 hours (~2 weeks) to identify (taking into account that sequencing identification is only performed once a week in the clinical mycology laboratory). Of the methods used after DTM MALDI, sequencing resulted in the most identifications (16%, 14/134), more than all the other methods combined. However, identification by sequencing took more than twice as long as these methods. In our study there were 30 organisms (22%) that were unidentified by MALDI-TOF after two rounds of DTM and two rounds of broth culture MALDI-TOF (**Table 2**). The majority (50%, 15/30) of these cases were due to species not being in the database. Another subset was not identified by MALDI-TOF due to inconclusive MALDI scores with mixed species IDs requiring confirmatory testing by slide culture or sequencing (27%, 8/30). There were also four cases of incorrect identifications (13%, 4/30) including two *Aspergillus sydowii* isolates incorrectly called *Aspergillus versicolor* and two *Trichophyton violaceum* incorrectly called *Trichophyton rubrum*. The remaining cases were two *Cladosporium* spp. and single *Sporothrix schenckii* that did not produce sufficient spectra for MALDI identification.

**Table 2:** Hours to identification from initial colony observation.

Avg in Hrs. (n)	1st DTM MALDI	2nd DTM MALDI	1st Broth MALDI	2nd Broth MALDI	No ID by MALDI	
					Slide Culture/Phenotypic	Sequencing
**All Organisms** (n=134)	**28** (74)	**70 **(24)	**144 **(5)	**138 **(3)	**96 **(8)	**381 **(22)
**Hyaline Hyphomycetes** (n=85)	**29 **(57)	**34 **(15)	**246 **(1)	**138 **(3)	**-**	**388 **(9)
* Aspergillus fumigatus* (n=25)	**23 **(22)	**51 **(3)	–	–	–	–
* Aspergillus montevidensis* (n=1)	–	**170 **(1)	–	–	–	**245 **(1)*
* Aspergillus niger* (n=17)	**21 **(12)	**32 **(4)	**246 **(1)	–	–	–
* Aspergillus sydowii* (n=3)	**51 **(1)	–	–	–	–	**438 **(2)
* Aspergillus terreus* (n=1)	–	**22 **(1)	–	–	–	–
* Aspergillus versicolor* (n=4)	**61 **(2)	–	–	**157 **(2)	–	–
* Cephalotrichum species^a,b^* (n=1)	–	–	–	–	–	**558 **(1)
* Cryptendoxyla hypophloia^b^* (n=1)	–	–	–	–	–	**431 **(1)
* Fusarium incarnatum equiseti complex* (n=1)	–	–	–	**98 **(1)	–	–
* Fusarium proliferatum* (n=1)	–	**24 **(1)	–	–	–	–
* Fusarium solani* (n=7)	**109 **(4)	**33 **(3)	–	–	–	–
* Fusarium solani species complex* (n=2)	–	**26 **(2)	–	–	–	–
* Paecilomyces variotii* (n=2)	**38 **(2)	–	–	–	–	–
* Penicillium brevicompactum* (n=1)	–	–	–	–	–	**102 **(1)
* Penicillium species^c^* (n=11)	**21 **(10)	**27 **(1)	–	–	–	–
* Phialemonium obovatum^b^* (n=1)	**-**	**-**	**-**	**-**	**-**	**176 **(1)
* Purpureocillium (Paecilomyces) lilacinum* (n=4)	**8 **(4)	–	–	–	–	–
* Scopulariopsis species^a,b^* (n=2)	–	–	–	–	–	**447 **(2)
**Dermatophytes** (n=22)	**26 **(9)	**90 **(5)	**121 **(1)	**-**	**88 **(4)	**418 **(3)
* Microsporum canis* (n=1)	**50 **(1)	–	–	–	–	–
* Trichophyton interdigitale* (n=2)	**8 **(1)	–	–	–	–	**171 **(1)
* Trichophyton rubrum* (n=8)	**59 **(2)	**90 **(5)	–	–	**174 **(1)	–
* Trichophyton tonsurans* (n=9)	**12 **(5)	–	**121 **(1)	–	**60 **(3)	–
* Tricohphyton violaceum* (n=2)	–	–	–	–	–	**541 **(2)
**Dematiaceous** (n=16)	**19 **(4)	**387 **(1)	**-**	**-**	**99 **(3)	**413 **(8)
* Alternaria alternata* (n=1)	**1 **(1)	–	–	–	–	–
* Acrophialophora levis^b^* (n=2)	–	–	–	–	–	**575 **(2)
* Aureobasidium pullulans* (n=1)	–	–	–	–	–	**308 **(1)
* Cladosporium species* (n=2)	–	–	–	–	**86 **(2)	–
* Exophiala dermatitidis* (n=3)	**12 **(2)	**387 **(1)	–	–	–	–
* Exophiala lecanii-corni^b^* (n=1)	–	–	–	–	–	**207 **(1)
* Lomentospora prolificans* (n=1)	**49 **(1)	–	–	–	–	–
* Phialophora species^a,b^* (n=1)	–	–	–	–	–	**412 **(1)
* Pleomonodictys species^a,b^* (n=1)	–	–	–	–	–	**389 **(1)
* Scytalidium cuboideum^b^* (n=1)	–	–	–	–	–	**388 **(1)
* Thermoascus species^a,b^* (n=1)	–	–	–	–	–	**386 **(1)
* Verruconis gallopava^b^* (n=1)	–	–	–	–	**125 **(1)	–
**Mucorales** (n=8)	**17 **(4)	**72 **(1)	**118 **(3)	**-**	**-**	**-**
* Mucor circinelloides* (n=6)	**19 **(3)	**-**	**118 **(3)	–	–	–
* Rhizopus arrhizus* (n=2)	**10 **(1)	**72 **(1)	–	–	–	–
**Basidiomycetes** (n=2)	**-**	**-**	**-**	**-**	**120 **(1)	**244 **(2)
* Anthracocystis flocculosa^b^* (n=1)	**-**	**-**	**-**	**-**	**-**	**313 **(1)
* Basidiomycete^b^* (n=1)	**-**	**-**	**-**	**-**	**120 **(1)	**-**
**Dimorphic**	**-**	**-**	**-**	**-**	**-**	**240 **(1)
* Sporothrix schenckii* (n=1)	**-**	**-**	**-**	**-**	**-**	**240 **(1)

a A definitive species level identification cannot be achieved by DNA sequencing.

b Species not included in database.

c Species correctly called by MALDI but was not validated at the time of the study. *This test was performed to validate MALDI result for an uncommon species, which was correct by MALDI.

The bolded values are the average times to identification from initial colony observation, in hours.

## Discussion

Despite the expected phenotypic and growth heterogeneity inherent to solid media culture, the direct transfer method demonstrated the most promise for improving TATs as the majority (73%, 98/134) of isolates could be identified by two rounds of DTM MALDI-TOF in under three days post-colony observation. Broth MALDI was only able to identify an additional 8 (6%, 8/134) of the isolates but at the cost of an additional 3 days to TAT.

The hyaline hyphomycetes were the most readily identified by MALDI-TOF MS resulting in 89% (76/85) of isolates being identified by MALDI-TOF MS overall and the greatest number of isolates identified in the first DTM MALDI-TOF MS step (43% 57/134). Many of these organisms are frequently encountered by laboratories (Luethy and Zelazny, 2018) and generate quality MALDI-TOF MS spectra with most extraction methods, allowing them to be more readily included in databases (Schulthess et al., 2014). While MALDI-TOF correctly identified all *Penicillium* sp. (n=10) to the species level, we had not yet validated species level identification, so we were only able report them to the genus level. There was also a case of *Aspergillus montevidensis* that was identified correctly by MALDI-TOF at the second DTM, but was ultimately sequenced in order to confirm the result of the uncommon species. In our study, the dermatophytes were also identified at high rates by MALDI-TOF MS (68% 15/22). All of the dermatophyte species we encountered in our study were in the database. However, the dermatophytes were the only group in our study that had isolates of species that were identified by MALDI and also had isolates of the same species that failed to be identified by MALDI. Many of the unidentified isolates produced inconclusive MALDI results so they had to be confirmed by slide culture. *Trichophyton violaceum* (n=2) was the only dermatophyte that was not able to be identified by MALDI despite being in the database.

The dematiaceous fungi, basidiomycetes, and dimorphic fungi were the most challenging to identify *via* MALDI-TOF MS. Only 31% (5/16) of dematiaceous fungi and none of the basidiomycetes or dimorphs were able to be identified with this method. For the basidiomycetes, this was due to a lack of database entries for these organisms. Dimorphs are also underrepresented in the database but that was not the case for the *Sporothrix* schenckii, as it is the only dimorphic fungi in the database. In the case of dematiaceous organisms, this is not unexpected as studies have reported challenges achieving sufficient spectra for identification with these organisms (Schulthess et al., 2014). Most basidiomycetes have not been reported to cause human infections and are generally considered contaminants when recovered in fungal culture. Working with dimorphic fungi requires enhanced safety precautions which makes adding them to databases more challenging.

Of the Mucorales, 38% were identified by MALDI-TOF MS within 24 hours and 75% of them in under 4 days from initial colony observation. Only two species were identified in our study, *Mucor circinelloides* and *Rhizopus arrhizus*. Both species had isolates that were identified in the first DTM MALDI and both had isolates that went as far as the full broth extraction method for identification. Infections with these organisms tend to progress very rapidly, making timely diagnosis and initiation of treatment critical to patient outcomes (Zhang et al., 2021).

Sequencing was the methodology that identified the most organisms that failed to be identified by MALDI-TOF, at nearly a 3-1 ratio with slide culture/phenotypic methods. Although the TATs for the sequencing took an average of 2 weeks, this could be improved by increasing the number of sequencing runs performed per week. However, this process is relatively labor intensive and requires a significant amount of training/expertise. For these reasons more frequent sequencing runs are not feasible in our laboratory but could be advantageous for other laboratories. As sequencing technology becomes faster and cheaper, it will certainly play an important role in the identification of filamentous molds.

To improve the utility of MALDI-TOF MS for filamentous molds identification, further attention should be paid to development of centralized databases that include spectra obtained from different growth conditions. This would allow for identification of molds with the added flexibility of using isolates that are cultured outside very rigid culture conditions to obtain reliable results. It would also be beneficial to not only supplement manufacturer databases with spectra obtained from clinical isolates, but to also develop data sharing and curation infrastructure to improve libraries and reduce duplication of efforts by different laboratories. Any attempts to centralize databases will also require the robust reporting of metadata (i.e., culture conditions, instrumentation used to generate spectra, and geographic location) to aid in using appropriate spectra for analysis (Zhang et al., 2021).

Overall, our study demonstrates a significant opportunity to leverage MALDI-TOF MS for the identification of clinical filamentous molds in the mycology laboratory. While there is still more work to be done to expand current databases, what is readily available still adds a significant benefit to TATs.

## Data Availability Statement

The raw data supporting the conclusions of this article will be made available by the authors, without undue reservation.

## Author Contributions

CH-S, WM, and SZ contributed to conception and design of the study. CH-S and WM performed testing. CH-S recorded data. CH-S and JF organized database and performed analysis. JF wrote the first draft of the manuscript. All authors contributed to manuscript revision, read, and approved the submitted version.

## Conflict of Interest

The authors declare that the research was conducted in the absence of any commercial or financial relationships that could be construed as a potential conflict of interest.

## Publisher’s Note

All claims expressed in this article are solely those of the authors and do not necessarily represent those of their affiliated organizations, or those of the publisher, the editors and the reviewers. Any product that may be evaluated in this article, or claim that may be made by its manufacturer, is not guaranteed or endorsed by the publisher.
